# Assessment of Major Animal Health Problems and Their Impact on Beef Cattle Production in Doba District of West Harerghe Zone, Ethiopia

**DOI:** 10.1155/2021/5533398

**Published:** 2021-08-23

**Authors:** Umer Seid Geletu, Ahmedin Abdureman Musa, Sisay Lemma Waqe, Munera Ahmednur Usmael, Yesihak Yusuf Mummed, Fufa Dawo Bari, Abdulmuen Mohammed Ibrahim

**Affiliations:** ^1^College of Agriculture, Department Animal Science, Oda Bultum University, P.O. Box 226, Chiro, Ethiopia; ^2^Oromia Bureau Livestock and Fishery Resources, West Hararghe Zone, Chiro Wereda, P.O. Box 226, Chiro, Ethiopia; ^3^School of Animal Science and Randge Land, Haramaya University, Dire Dawa, Ethiopia; ^4^Department of Microbiology, Immunology and Veterinary Public Health, College of Veterinary Medicine and Agriculture, Addis Ababa University, P.O. Box 34, Bishoftu, Addis Ababa, Ethiopia; ^5^Office of Research Affairs, Haramaya University, Dire Dawa, Ethiopia

## Abstract

The aim of the current study was to assess the major animal health problems and their impact on beef cattle production in Doba district of West Harerghe Zone, Ethiopia. The study area was purposively selected, and a simple random sampling method was used to selected households' fatteners from each kebele and interviewed using structured questionnaires. The present study showed that the overall prevalence of the diseases was internal and external parasite 93.3%, bloat 53.3%, black leg 71.1%, pasteurolosis 71.8%, wound 71.8%, FMD 22.2%, and anthrax 13.33% which affect fattening cattle, respectively, in the study area. All the respondents (100%) involved in the study were experienced with deworming of their animals to protect from parasites. But, only 46.7% and 42.2% of the respondents have accessed veterinary services with limited regularity and vaccination program, respectively, in the study area. Hence, the beef cattle fatting sector should be supported through considering alleviating the major disease affecting this sector and encouraging the farmers' indigenous knowledge practice with technology.

## 1. Introduction

The major constraints in fattening cattle production have been health problems because of the decrease of production, slow rate of regeneration, and amplification of the risk of disease transmission [1]. Infectious animal diseases that are endemic generate a variety of significant impact economically such as mortality, morbidity. Diseases have numerous negative impacts on productivity and fertility of herds [2]. Thus, knowing the status of major problems that constrain beef cattle fattening can help improving productivity and market success of producers; with the purpose of contributing in poverty reduction at all through market-oriented agricultural development [3].

Traditional backyard cattle fattening is widely practiced in highland areas. This type of cattle fattening was almost entirely dependent on locally available resources to minimize costs of fatting. In areas such as Hararghe, farmers buy young oxen from the nearer lowland area and use them for ploughing for a couple of years after which they fatten and sell them before they become old and emaciated [4].

The well-known benefits of conducting biosecurity for control and prevention of disease are improved efficiency of productivity [5, 6], keeping good welfare of the animals, boosting the response of immune systems to vaccines, and increased job satisfaction for producers [7]. Biosecurity practices were recommended by a number of studies based on different production systems. Almost all of the studies prefer the use of preventive procedures, but they do not often provide evidence on cost effectiveness [8].

Alhough Ethiopia is known for the largest cattle population, most beef was produced under an extensive production system, with low input system as a result of which beef production and productivity are very poor as compared to the world beef production. So far, there is no documented information regarding major animal health problems in Doba district of West Hararghe Zone of Eastern Ethiopia.

The general objective of the current study was

- To assess the major animal health problems and their impact on beef cattle production in Doba district of West Harerghe Zone, Eastern Ethiopia

The specific objective of the study was

- To identify major health problems of beef cattle in the study area and their associated risk factors

## 2. Materials and Methods

### 2.1. Descriptions of the Study Area

**Figure 1:**
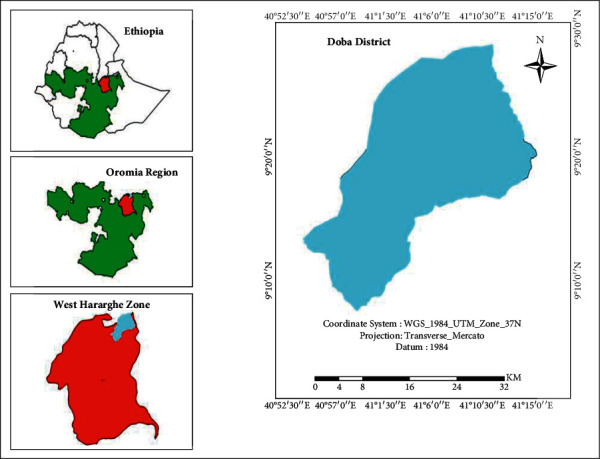
Map of the study area (source: the author).

This study was conducted in selected kebeles of Doba district, West Harerghe Zone, Ethiopia (Figure 1). Doba district has 133,939 total populations of whom 68,512 were male and 65,427 were female and area coverage about 729 km^2^ or 72,900 hectares according to the census in 2007. It is one of the 17 districts in West Hararghe Zone, and it is located around 383 km east of Addis Ababa. Geographically, it is situated between 9° 0′to 9° 30′ latitude and 40°57′ to 41°15′ longitude. Altitude ranges from 1,149 to 2,733 meters above sea level. The average temperature ranges from 21°C to 28°C. The annual rainfall ranges from 650 to 750 mm. The majority of the population is Muslim (86%), and the primary language spoken is Afan Oromo [9].

### 2.2. Sampling Size and Sampling Techniques

#### 2.2.1. Selection of the Study Area

Doba district was purposively selected based on nearer to main road and climatic condition. Accordingly, three kebeles from doba district, namely, *Urji Berisa, Ifa Aman, and Gemechu* were randomly selected. From each kebele, 15 cattle fatteners or owner was selected by the simple random sampling technique, and a total of 45 households were included in the current study and interviewed using structured questionnaires.

### 2.3. Data Collection and Analysis

A questionnaire was designed to get all information related to the major beef cattle fatting health problem in the study area. Agricultural experts, development agents, and veterinarians were key informant interviewees. Data were coded and entered into a Microsoft excel and analyzed using *R* software. The analysis and summarization of the data was made using descriptive statistics.

## 3. Results

The overall respondents of cattle fattener showed that most of the interviewees (86.7%) were male and the others were female (13.3%) (see Table 1).

### 3.1. Occurrence of Diseases

The overall interviewed cattle fatteners (100%) from the three kebeles reported the occurrence of animal health problems, especially parasites (internal and external) which affect beef cattle fattening (see Table 2). Out of the totally interviewed cattle fatteners, 41.66% of them have reported that they have experienced beef cattle loss due to death which directly affected their economy.

### 3.2. Control Measures against Diseases

All cattle fatteners (100%) in the three kebeles have been practicing the deworming program to protect their beef cattle from internal and external parasites. However, only 53.33% and 41.66% of the respondents were accessed to veterinary services and vaccination practice in the study area (see Table 2).

Cattle fattening owners were also asked to describe major diseases, which affect beef cattle in the study area, and prioritize them based on their relative degree of importance. Respondents described diseases in their local names (see Table 3). These local names were given their veterinary equivalent name based on the symptoms mentioned and discussions with veterinarians in the area.

## 4. Discussion

According to the current study, the majority of respondents said that diseases are one of the most common limiting factors of the beef cattle fattening system in the area. In addition, they indicated that the disease was aggravated by a number of factors such as lack of veterinary service, lack of awareness of the society towards disease prevention and control program, and lack of attention to animal health from the government. Animal health professionals in the district have also explained that animal diseases are one of the main problems in the area, but there is a scarcity of drugs and vaccines for the treatment of their livestock due to lack of transport. Furthermore, the respondents reflected that they need to have the knowledge of how to improve their fattening cattle health condition by having access to drugs and some important vaccines.

The study showed that parasites were the most common health problems of cattle fatting in all three kebeles of the study area. Only 42.2% of cattle fatteners have vaccinated cattle against some of the diseases while entering the feedlot. This shows that a majority of the cattle fatteners in the study site do not know how to prevent disease in their farm before disease occurrence. Only 46.7% of the cattle fatteners involved in the study reported access to veterinary services from government organizations.

Black leg, bloat, pasteurelosis, and FMD (foot and mouth disease) are the most common infectious animal diseases of cattle fattening practice in Doba district (Table 3). Out of the total 45 respondents interviewed, 71.1% of them reported that it is the main disease challenging beef cattle production. This was in agreement with that described by Radiostits [10].

In the study area, the respondents reported that in 46.7%, the FMD case affects their cattle fattening practice. FMD causes lower rates of live-weight gain in beef cattle production due to reduced feed intake [11]. The study revealed that anthrax is also the disease affecting beef cattle in the district because only 13.33% of the overall respondents reported the anthrax case in their cattle fattening practice.

The case of bloat in fattening beef cattle was common in the Doba district. Majority of the respondents (53.3%) involved in the study reported that the bloating case is common.

## 5. Conclusions and Recommendation

The major constraint of beef cattle fattening production is the health problem due to decrease of production, slow rate of regeneration, and amplification of the risk of disease transmission. The overall interviewed cattle fatteners (100%) from the three kebeles reported the occurrence of animal health problems, especially parasites which affects fattening cattle. The study indicated that the overall interviewed cattle fatteners reported the presence of diseases such as internal and external parasite, bloat, black leg, pasteurolosis, wound, FMD, and anthrax which affect fattening cattle by (93.3%, 53.3%, 71.1%, 71.8%, 71. 8%, 22.2%, and 37.8%), respectively, in the study area. All the respondents (100%) involved in the study have a practice of deworming to protect cattle from internal and external parasites. But, only 46.7% and 42.2% of respondents have accessed veterinary services with limited regularity and vaccination program, respectively, in the study area. Out of the totally interviewed cattle fatteners, 38.7% of them have reported that they have experienced animal beef cattle loss due to death which directly affected their economy.

Depending on the study, the following recommendations are forwarded:

- Beef cattle fatting owners should receive basic training regarding control and prevention animal diseases
- Regular vaccination program against major animal diseases (blackleg, pasteurellosis, FMD, and anthrax) should be practiced
- A proper animal health delivery system that could be extended to all livestock owner should be developed

## Figures and Tables

**Table 1:** Demographic features of the respondents.

Respondent's	N	Category	Frequency	% (n)
Sex	45	Male	39	86.7
		Female	6	13.3

Educational status	45	Illiterate	30	66.7
		Literate	11	24.4
		Primary school	4	8.9

Family age size	45	<15 years	17	37.8
		≥15 years	28	62.2

**Table 2:** Occurrence of diseases and producers' access to animal health protection facilities in the selected three kebeles of Doba district.

Parameters	Ifa Aman % (*n*)	Urji Berisa % (*n*)	Gemechu % (*n*)	Total % (N)
Occurrence of health problems				
Yes	100 (15)	100 (15)	100 (15)	100 (45)
No	0.0 (0)	0.0 (0)	0.0 (0)	0.0 (0)

Vaccination practice				
Yes	33.3 (5)	40 (6)	53.3 (8)	42.2 (19)
No	66.7 (10)	60 (9)	46.7 (7)	57.8 (26)

Deworming practice				
Yes	100 (15)	100 (15)	100 (15)	100 (45)
No	0.0 (0)	0.0 (0)	0.0 (0)	0.0 (0)

Access to veterinary services				
Yes	46.7 (7)	40 (6)	53.3 (8)	46.7 (21)
No	53.3 (8)	60 (9)	46.7 (7)	53.3 (24)
Animal loss due to diseases				
Yes	53.3 (8)	33.3 (5)	26.7 (4)	37.8 (17)
No	46.7 (7)	66.7 (10)	73.3 (11)	62.2 (28)

**Table 3:** Major diseases affecting beef cattle in the selected three kebeles of Doba district.

Diseases	Local name		Ifa Aman % (*n*) (*n* = 15)	Urji% (*n*) (*n* = 15)	Gemechu % (*n* = 15)	Total % (N), *N* = 45
External parasites	Raammoo	Yes	86.7 (13)	100 (15)	93.3 (14)	93.3 (42)
		No	13.3 (2)	0.0 (0)	6.7 (1)	6.7 (3)

Black leg	Abbaa Gorbaa	Yes	80 (12)	73.3 (11)	60 (9)	71.1 (32)
		No	20 (3)	26.7 (4)	40 (8)	28.9 (13)

Pasteurolosis	Gorora	Yes	86.7 (13)	80 (12)	46.7 (7)	71.1 (32)
		No	13.3 (2)	20 (3)	53.3 (8)	28.9 (13)

Bloat	Bokoka	Yes	40 (6)	66.7 (10)	53.3 (8)	53.3 (24)
		No	60 (9)	33.3 (5)	46.7 (7)	46.7 (21)

Anthrax	Abbaa sangaa	Yes	20 (3)	13 (2)	6 (1)	13.3 (6)
		No	80 (12)	86.7.3 (13)	93.3 (14)	86.7 (39)

Wound	Madaa	Yes	20 (3)	33.3 (5)	13.3 (2)	22.2 (10)
		No	80 (12)	66.7 (10)	86.7 (13)	77.8 (35)

FMD	Maasa	Yes	60 (9)	33.3 (5)	46.7 (7)	46.7 (21)
		No	40 (6)	66.7 (10)	53.3 (8)	53.3 (24)

## Data Availability

The data used to support the findings of this study are available from the corresponding author upon request.
